# A Rare Presentation of Erythrodermic Psoriasis

**DOI:** 10.7759/cureus.75105

**Published:** 2024-12-04

**Authors:** Kriti Vaidya, Kunam Mukan, Lucy Sharpe, Lindsay Whittam, Chris Jacobs

**Affiliations:** 1 Internal Medicine, Great Western Hospital, Swindon, GBR; 2 Medical Photography, Great Western Hospital, Swindon, GBR; 3 Dermatology, Great Western Hospital, Swindon, GBR; 4 Psychology, University of Bath, Bath, GBR; 5 Postgraduate Medical Education, Great Western Hospitals NHS Foundation Trust, Swindon, GBR

**Keywords:** erythrodermic psoriasis, erythrodermic psoriasis (ep), psoriasis, psoriasis area and severity index (pasi), psychosocial stress

## Abstract

Erythrodermic psoriasis (EP) is a rare and challenging-to-diagnose severe form of psoriasis. Its presentation can have similarities with other inflammatory skin conditions, complicating subsequent management. We present a case of a 76-year-old woman with EP who presented with fever, tachycardia, leg swelling with pain and redness, and reduced consciousness. Her imaging revealed characteristic features of the disease, providing valuable educational insights. We aim to emphasize the importance of maintaining a high index of suspicion for EP and promptly adjusting treatment.

## Introduction

Erythrodermic psoriasis (EP) is a rare and severe form of psoriasis vulgaris, with a 1-2.25% prevalence among psoriatic patients [1]. It is defined by considerable erythema that covers at least 80-90% of the body surface [2] and is frequently associated with fever, chills, headache, and general discomfort [3]. EP often mimics a burn-like appearance of the skin due to the intense inflammation, and the associated systemic symptoms can make it difficult to differentiate from other severe skin conditions.

The onset might be abrupt or gradual and can be triggered by a range of factors, such as mental stress, infections, or new medications [4]. The pathophysiology of EP requires additional research; however, certain immunological biomarkers, like interleukin-4, interleukin-10, IgE antibodies, and T-helper 2 lymphocytes, are suspected to be implicated. EP is a medical emergency because it disrupts the skin’s normal barrier functions, which are crucial for regulating body temperature, preventing infections, and maintaining fluid and electrolyte balance. The loss of these protective functions can lead to severe fluid loss, multiorgan failure, and an increased risk of infections. The management of EP can be very challenging, and if left untreated, the patient could be at risk of experiencing multi-system organ failure and high-output heart failure due to cutaneous volume loss [5-7].

We present a case where EP is evident in a patient, serving as a manifestation of recent psychological stress on a background of multiple treatment failures. The extent of the disease in this report was measured using the Dermatology Life Quality Index (DLQI). DLQI is a questionnaire comprising 10 questions aimed at assessing the influence of skin conditions on an individual's quality of life. Each question receives a score between 0 and 3, resulting in a total score range from 0 (indicating no effect of the skin condition on quality of life) to 30 (suggesting the highest impact on quality of life) [8].

## Case presentation

A 76-year-old woman with poorly controlled psoriasis (DLQI 28) was referred to the medical expected unit after experiencing generalized weakness with limited movement, fever, tachycardia, leg swelling associated with pain and redness, decreased appetite, and a reduced level of consciousness. The patient had recently visited her general practitioner for leg cellulitis, for which she was given oral antibiotics that were ineffective.

She has been struggling with psoriasis and psoriatic arthritis for about 30 years, for which she was under both dermatology and rheumatology specialists. She had tried various disease-modifying anti-rheumatic drugs, notably sulfasalazine, leflunomide, and cyclosporin, which provided marginal benefits. The patient is presently undergoing treatment with 25 milligrams (mg) of subcutaneous methotrexate administered weekly. Additionally, it has been observed that she recently experienced the loss of her daughter, an event that may have exerted a psychosocial impact on her.

**Figure 1:**
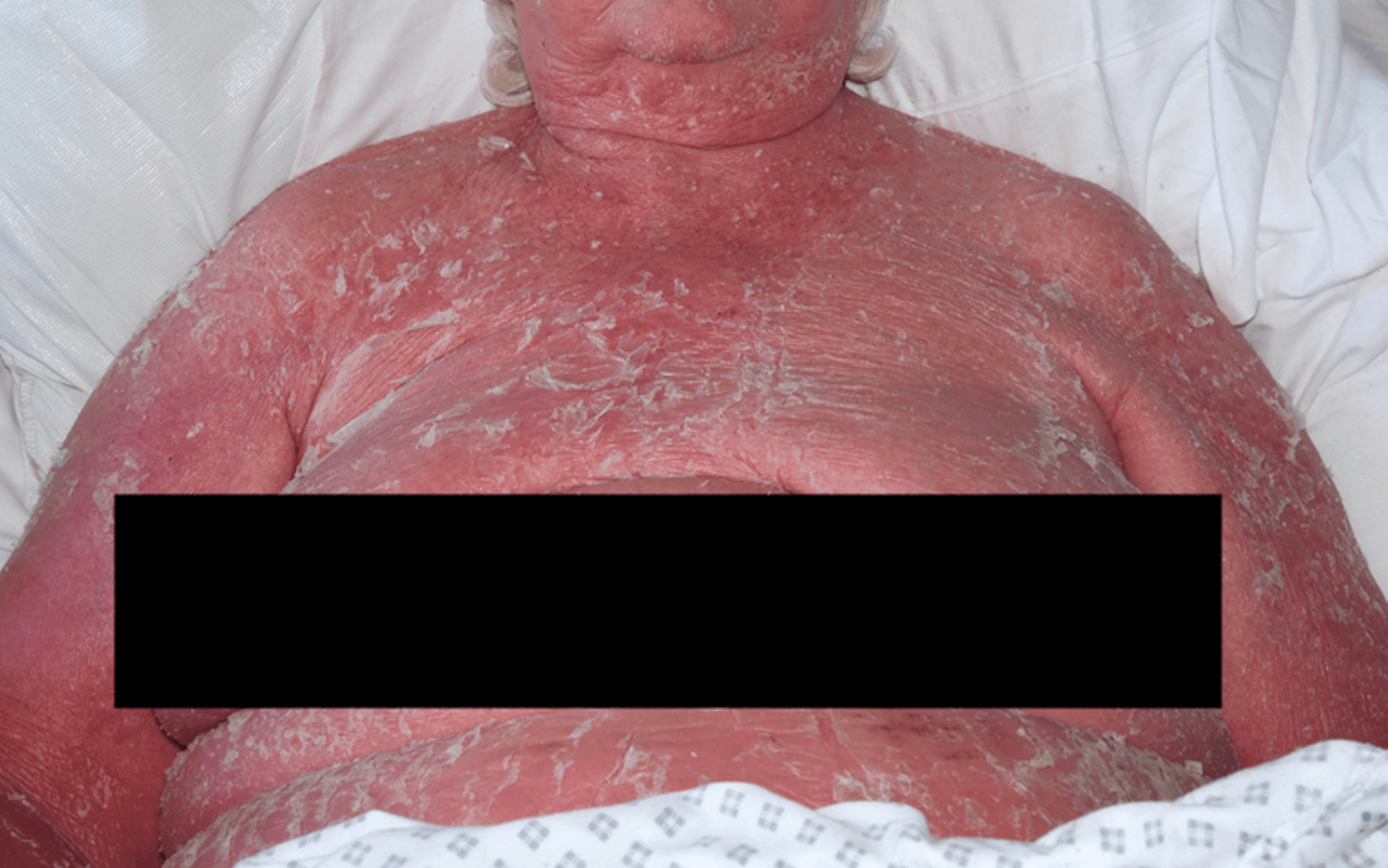
Front view

**Figure 2:**
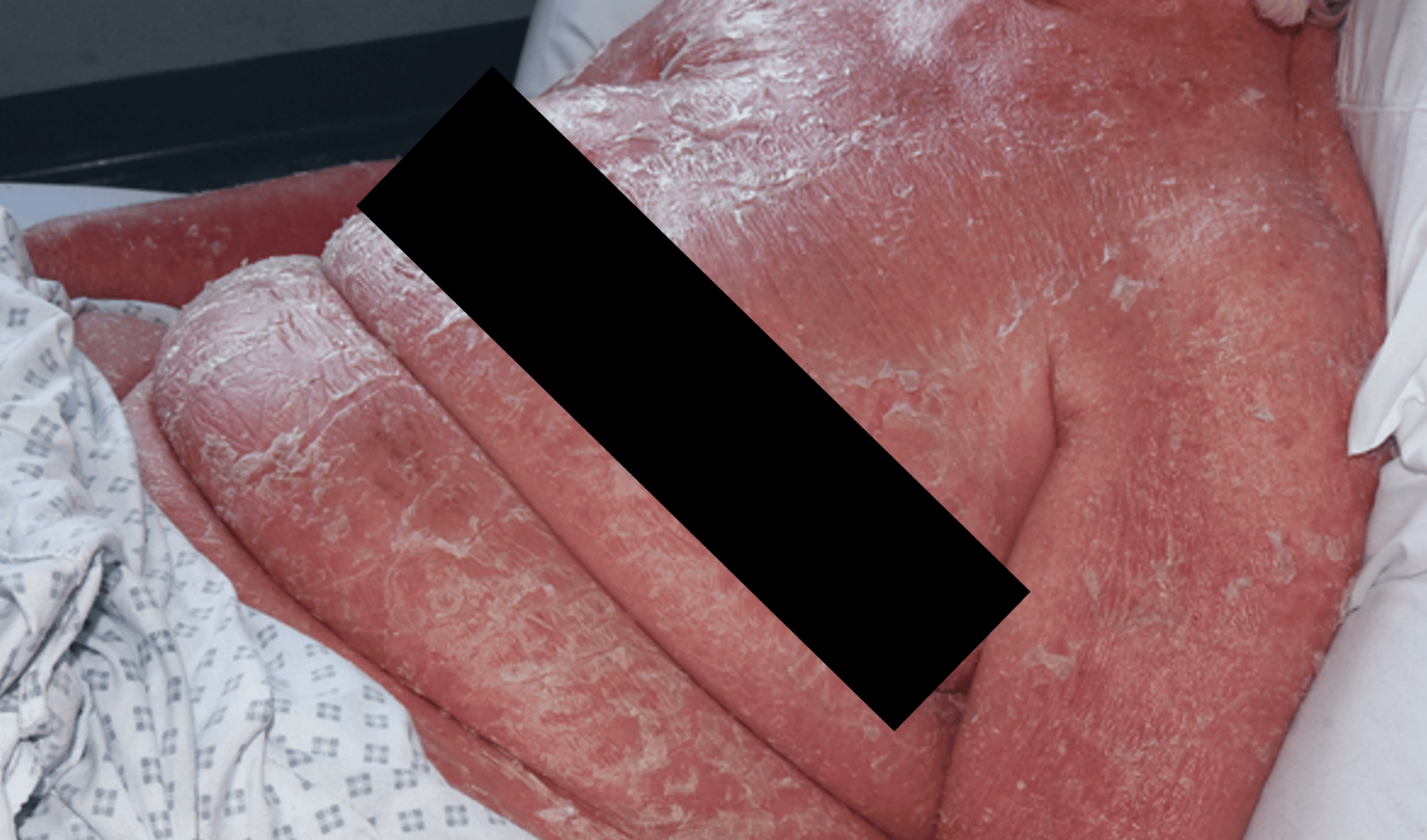
Profile view

**Figure 3:**
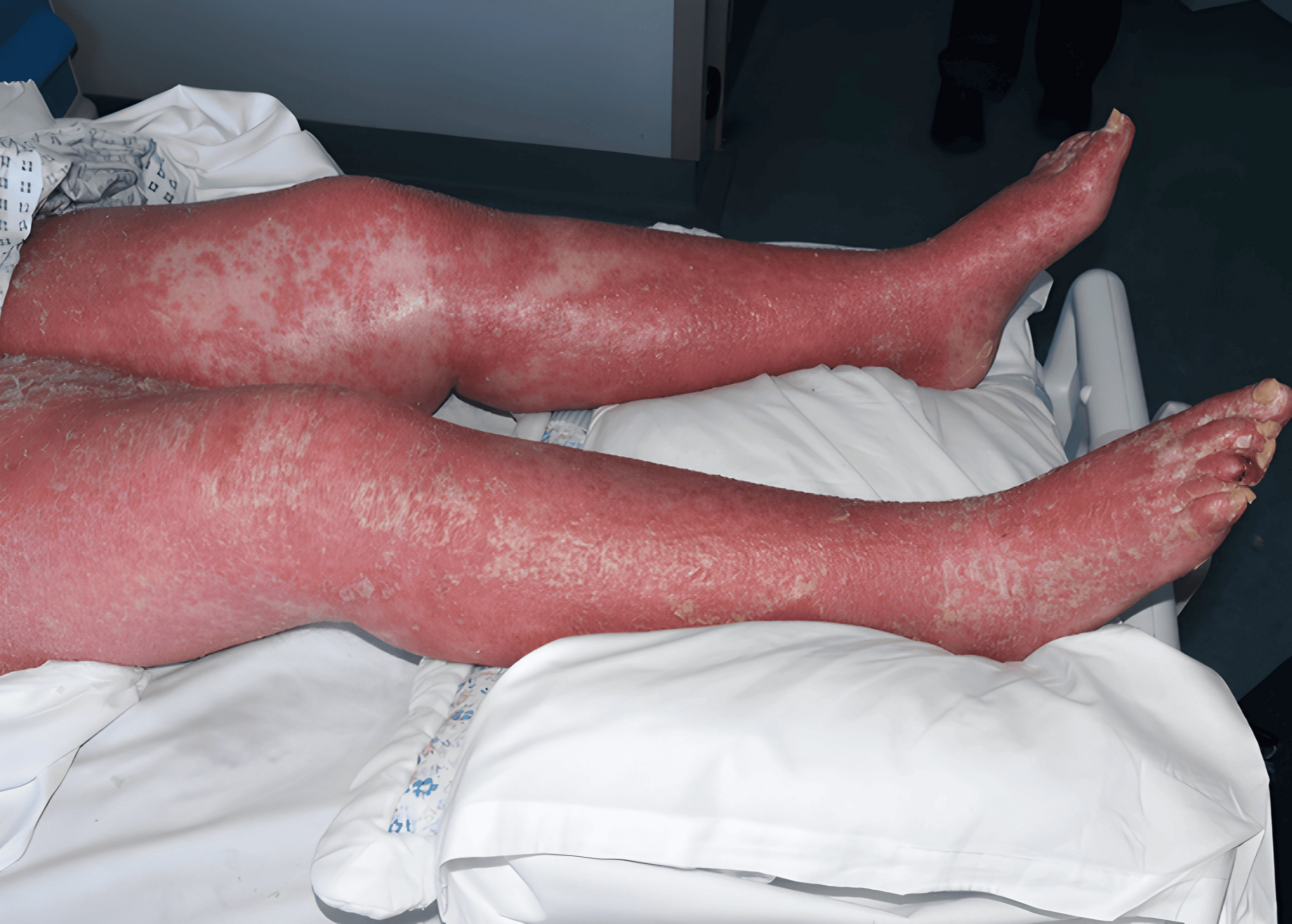
Leg edema

During the clinical assessment, psoriasis was observed to involve the entire body surface area, accompanied by a low-grade fever. The skin was warm to touch, dry, and thickened with prominent silvery-white scaling (Figures 1-2). Notably, there was substantial edema in the lower extremities with localized cellulitis, resulting in restricted mobility (Figure 3). In the hospital setting, the patient was initially treated with intravenous clindamycin at a dose of 600 mg four times daily for two days, after which therapy was switched to oral clindamycin, and the patient subsequently remained afebrile. Methotrexate was withheld while treating the acute infection, and the patient was started on adalimumab following a short course of intravenous hydrocortisone. The results of the blood tests during admission indicated an improvement in C-reactive protein and normalization of serum creatinine following fluid management (Table 1). Mild thrombophilia had also improved after discharge from the hospital, and neutrophilia was observed to be related to steroid initiation.

**Table 1:** Lab results of the patient highlighting the blood test before and after treatment eGFR-EPI: estimated glomerular filtration rate using the chronic kidney disease epidemiology collaboration (CKD-EPI) equation

	On admission	On discharge	Units	Reference range
Total white blood cells	11.8	16.0	x10⁹/L	4-10
Neutrophils	7.58	12.00	x10⁹/L	2-7
Platelets	552	492	x10⁹/L	150-400
C-reactive protein	97	29	mg/L	0-5
eGFR-EPI (per 1.73 m²)	52	67	mL/min	59-9999
Serum creatinine	92	75	µmol/L	45-84

The effectiveness of adalimumab was re-evaluated after a period of six weeks. Notable improvement in the skin symptoms was observed. Nevertheless, the patient continued to exhibit some visible signs of the disease, and hence, another assessment of adalimumab's efficacy will be conducted at 16 weeks.

Photography

Photography of this patient was quite restricted due to her condition and lack of mobility. However, with nursing help and patient compliance, a variety of different views were obtained to illustrate the condition. There was plenty of natural light available in the room, so an ISO (film speed) of 3200 was used with some additional fill-in-flash to enhance the texture and detail of the condition within the images (Figures 1-3).

## Discussion

Psoriasis (psoriasis vulgaris) is a chronic (long-term) autoimmune condition characterized by extensive scaling caused by epidermal cell hyperproliferation. It typically presents with large oval-circular plaques over the scalp, trunk, and extensor body surface [9]. More commonly, EP arises as a complication of psoriasis vulgaris [10].

Although EP is classified as a subtype of psoriasis, the pathogenic processes are distinct from those seen in plaque psoriasis [11], IL36RN mutations have been linked to pustular psoriasis, and the class I antigens HLA-Cw6, HLA-B57, HLA-B13, and HLA-B17 have been linked to psoriasis vulgaris. However, very little is known about the genetic foundation of EP [12]. Several studies suggest that the disease is associated with a predominantly T-helper 2 phenotype.

In this report, the patient’s symptoms were classic EP, including widespread erythema, desquamation, and systemic involvement. Notably, the patient also developed cellulitis of the lower limbs (Figure 3). Given the severity of the infection, the cellulitis was treated with antibiotics to prevent further complications, such as sepsis, which can occur if left untreated. The co-occurrence of an infection with EP underscores the delicate nature of the skin’s integrity in this condition.

A contributing factor to the relapse of EP in this patient might have been the psychological stress related to a significant life event that the patient described. Stress is well-known to trigger or exacerbate psoriasis by altering immune system activity and increasing inflammation.

The mechanism by which psychological stress contributes to the onset or worsening of psoriasis is not yet fully understood. Psychological stress activates the hypothalamic-pituitary-adrenal axis, leading to the release of stress hormones (cortisol, adrenaline, and noradrenaline) that interfere with immune regulation and amplify inflammatory responses, fostering a pro-inflammatory environment [13]. Although cortisol typically exerts anti-inflammatory effects, prolonged stress may diminish its regulatory capacity, allowing unopposed inflammatory pathways [14].

EP, in particular, is propelled by an immune response dominated by T-cells, particularly Th1 and Th17 subsets, along with dendritic cells that produce potent pro-inflammatory cytokines such as IL-17, IL-22, TNF-α, and IFN-γ [15]. Chronic stress further amplifies T-cell activation, significantly intensifying the immune response. This excessive cytokine production may result in a “cytokine storm,” a widespread and severe inflammatory reaction that manifests as extensive skin redness, scaling, and edema [16].

Additionally, EP compromises the skin’s barrier function, heightening the risk of infection and fluid loss. In cases triggered by stress, dysregulated cortisol levels may further undermine skin barrier integrity. Together with the high systemic inflammation, these factors render the skin highly susceptible to infections, while stress-related immune suppression increases the body’s vulnerability to opportunistic infections [17].

Nonpharmacological treatments, such as effective stress management techniques, mindfulness meditation, cognitive-behavioral therapy, relaxation techniques, and physical exercise, can help patients reduce their physiological stress response. In five out of six randomized control trials, participants demonstrated improvements in self-administered psoriasis area and severity index scores following eight or 12 weeks of meditation and/or mindfulness interventions. Additionally, two studies indicated psychological benefits for psoriasis patients after engaging in these practices. Collectively, these findings imply that meditation may serve as an effective method for enhancing both psoriasis severity and the quality of life for patients [18].

Pharmacological treatment usually includes oral hydration, topical steroids, vitamin D analogs, and a trial of biological therapy. In this instance, due to the severity of the condition and the risk of further systemic complications, the decision was made to initiate treatment with adalimumab, a TNF-alpha inhibitor. Adalimumab was chosen because of its potent anti-inflammatory properties, which specifically target the overactive immune response seen in psoriasis. It effectively neutralizes TNF activity by impeding its interaction with TNF receptors on the cell surface. This inhibition curtails the migration of leukocytes, subsequently reducing the proliferation and differentiation of keratinocytes [19,20].

In combination with adalimumab, the patient was also prescribed methotrexate, a well-known immunosuppressive medication that inhibits the rapid turnover of skin cells and reduces immune system activity. Methotrexate is often used alongside biologics to enhance treatment efficacy and to provide more comprehensive immune suppression.

## Conclusions

This case report represents a rare presentation of EP associated with psychological stress. When patients present critically ill with an erythrodermic rash, it is important to consider EP in the differential diagnosis, especially with recent psychological stress. The mechanisms through which psychological stress interferes with psoriasis onset or exacerbations are not completely understood. However, psychoneuroimmunology studies have shown that acute and chronic stress can affect immune function, leading to a worsening of psoriasis by inducing keratinocyte proliferation and incomplete maturation.
